# Population-Specific Salinity Tolerance in the Extremophile *Colobanthus quitensis*: Evidence of Adaptive Plasticity

**DOI:** 10.3390/plants14203116

**Published:** 2025-10-10

**Authors:** Marely Cuba-Díaz, Yadiana Ontivero, Eduardo Fuentes-Lillo, Macarena Klagges, Paulina Arriagada, Gustavo Cabrera-Barja, Benjamín Sepúlveda

**Affiliations:** 1 Laboratorio de Biotecnología y Estudios Ambientales (LABEA), Departamento de Ciencias y Tecnología Vegetal, Escuela de Ciencias y Tecnología, Universidad de Concepción, Campus Los Ángeles, Juan Antonio Coloma 0201, Los Ángeles 4440000, Chile; yadibiologa2019@gmail.com (Y.O.); macaklagges@gmail.com (M.K.); pauarriagada1992@gmail.com (P.A.); benjasepul2020@udec.cl (B.S.); 2 Programa de Ciencia Antártica y Subantártica (PCAS), Universidad de Concepción, Concepción Barrio Universitario s/n, Concepción 4030000, Chile; 3 Laboratorio de Invasiones Biológicas (LIB), Facultad de Ciencias Forestales, Universidad de Concepción, Concepción 4030000, Chile; efuenteslillo@gmail.com; 4 Instituto de Ecología y Biodiversidad (IEB), Concepción 4030000, Chile; 5 Facultad de Ciencias de la Rehabilitación y Calidad de Vida, Escuela de Nutrición y Dietética, Universidad San Sebastián, Campus Las Tres Pascuales, Lientur 1439, Concepción 4080871, Chile; gustavo.cabrera@uss.cl

**Keywords:** oxidative stress, osmolyte accumulation, antioxidant enzymes, abiotic stress

## Abstract

Salinity is a major abiotic stress that limits plant growth and survival. *Colobanthus quitensis*, the only native dicotyledon in the Antarctic Peninsula and southern South America, naturally inhabits environments with contrasting salinity regimes. This study compared the salt stress responses of three geographically distinct populations—Antarctic (pA), Magellanic coastal (pPA), and Andean inland (pC)—exposed to 0, 50, and 150 mM NaCl under controlled conditions. Morpho-physiological traits, photosynthetic parameters, osmolyte accumulation, oxidative damage markers, and antioxidant responses were evaluated. Population-specific strategies were observed. In pA, salinity reduced shoot biomass by 58% and doubled lipid peroxidation levels at 50 mM, indicating high oxidative stress. In pPA, shoot growth was maintained even at 150 mM, although chlorophyll and carotenoid contents decreased by approximately 20%, along with a reduction in total antioxidant capacity. In contrast, pC showed a coordinated tolerance response, maintaining biomass while accumulating the highest proline levels (742 µmol g^−1^ FW at 150 mM) and enhancing total antioxidant capacity by 35% compared to the control. Multivariate analyses supported the contrasting strategies among populations. These results provide novel evidence of local adaptation and ecological plasticity in *C. quitensis*, particularly highlighting the hidden resilience of non-coastal populations. The findings support the potential of this extremophile species as a model system for investigating salinity tolerance and as a promising genetic resource for developing biotechnological strategies aimed at improving crop resilience under saline conditions.

## 1. Introduction

Soil salinization is a major abiotic stressor that limits plant growth and productivity worldwide [1]. Recent estimates indicate that over 50% of agricultural lands are affected by salinity, resulting in significant crop losses and reduced food security [2,3]. By 2050, more than half of all arable land is projected to become salinized, primarily due to unsustainable irrigation practices, climate change-induced droughts, and the overuse of chemical fertilizers, which deposit excessive salts in the soil [4]. This underscores the urgency of understanding plant salt tolerance and identifying resilient genotypes for agriculture and ecosystem restoration.

Plant tolerance to salinity involves complex physiological mechanisms, including osmotic adjustment, ion compartmentalization, and oxidative stress mitigation. Salinity imposes both osmotic stress, which limits water uptake, and ionic stress, due to the accumulation of toxic ions such as Na^+^ and Cl^−^, leading to nutrient imbalance [1,5]. Among compatible solutes, proline and soluble sugars play a key role in maintaining cellular turgor and protecting metabolic integrity [5,6]. Salinity-induced oxidative stress increases the production of reactive oxygen species (ROS), which damage lipids, proteins, and nucleic acids. To survive these extreme conditions, plants also modulate stress-responsive gene expression and hormone signaling pathways, including abscisic acid (ABA), ethylene, and jasmonic acid, which orchestrate adaptive responses [1]. To counteract these effects, plants activate antioxidant systems, including enzymatic defenses such as superoxide dismutase (SOD), catalase (CAT), and peroxidases, as well as non-enzymatic compounds like phenolics and flavonoids [7,8]. These integrated responses allow plants to maintain cellular homeostasis, protect organelle function, and ensure survival under prolonged salinity stress. It is worth noting that the efficiency and predominance of these mechanisms can differ between halophytes, which are naturally adapted to high salinity, and glycophytes, which are more sensitive to salt stress [1].

Conventional breeding for salinity tolerance has achieved limited success due to the polygenic nature of this trait and its strong interaction with the environmental conditions. Consequently, the study of naturally tolerant plant species can provide valuable insights into adaptive strategies, which may support the development of biotechnological tools or inform breeding approaches [9,10].

*Colobanthus quitensis*, the only native dicotyledonous plant in Antarctica, exhibits a disjunct distribution from Mexico to the Antarctic Peninsula, inhabiting high-altitude and sub-Antarctic ecosystems along the Andes and southern South America [11,12]. This broad geographic range has led to pronounced phenotypic plasticity among populations, including differences in morphology (e.g., leaf size and flower characteristics), physiology (e.g., variability in freezing tolerance), and genetic structure (e.g., strong isolation-by-distance patterns and low gene flow between populations) [13,14]. Notably, some coastal populations are exposed to seawater flooding or marine spray [15,16], whereas high Andean populations grow in habitats without direct marine influence. This environmental heterogeneity offers a unique opportunity to investigate whether salinity tolerance is confined to coastal populations or whether inland populations exhibit cross-tolerance associated with other abiotic stresses, such as high radiation, low temperatures, and drought.

Although *C. quitensis* has been cited as a salinity-tolerant species, few studies have examined the physiological or biochemical mechanisms underlying this trait. Previous research has documented salinity responses during germination and in vitro propagation, focusing on germination rates and early morphological performance [17]. More recently, population-specific differences in the percentage and speed of in vitro germination under saline conditions have been reported [18]. Preliminary unpublished data from our research group (LABEA; M.C.D., personal communication) suggest population-specific responses at later developmental stages, a comprehensive analysis comparing the salinity responses of different populations using mature plants under common garden conditions remains lacking. Such an approach is crucial for evaluating the ecological relevance and consistency of tolerance mechanisms beyond early developmental stages, and for identifying population-level adaptive strategies under stress scenarios.

This study aims to address this knowledge gap by evaluating the morphological, physiological, and biochemical responses of three *C. quitensis* populations originating from habitats with contrasting environmental conditions: a salt marsh population subject to periodic seawater flooding (pPA), a coastal population exposed to marine spray (pA), and a high-elevation Andean population without marine influence (pC). We hypothesize that populations naturally exposed to salinity (pPA and pA) have evolved more efficient mechanisms to cope with salt stress, which will be reflected in enhanced growth performance, osmotic adjustment, and antioxidant defenses under saline conditions. In contrast, the inland Andean population (pC) is expected to display less coordinated or attenuated responses due to the historical absence of marine-derived salinity in its native environment.

To test this hypothesis, we addressed the following questions: (1) Do populations of *C. quitensis* differ in their morphological and physiological responses to salt stress depending on their native habitat? (2) Are there population-specific differences in osmolyte accumulation and antioxidant defense mechanisms under increasing salinity? (3) Do these differences reflect distinct adaptive strategies to saline versus non-saline environments? By addressing these questions under controlled conditions, this study positions *C. quitensis* as a valuable model for investigating salt stress adaptation in extremophile plants, with implications for understanding local adaptation, advancing ecological theory, and informing biotechnological strategies for crop improvement under climate change scenarios.

## 2. Results

### 2.1. Morpho-Physiological Responses to Salinity Stress 

Salinity exposure significantly affected the morphological traits of *C. quitensis*, revealing clear inter-population differences (Table 1). The Antarctic population (pA) exhibited the highest sensitivity to salinity. The number of leaves decreased by 38% under moderate salinity (from 18.67 ± 0.93 at 0 mM to 11.47 ± 1.02 at 50 mM NaCl; *p* < 0.05), with a further decline at 150 mM (8.87 ± 0.50). Leaf length was markedly reduced from 13.02 ± 0.63 mm (0 mM) to 8.86 ± 0.38 mm (150 mM NaCl). Root length decreased by 32% under the highest salinity (from 45.90 ± 2.35 mm to 31.15 ± 1.49 mm). A sharp reduction in total biomass (−58%) and water content (−13%) further indicated a strong structural and physiological impact of salinity stress.

In contrast, the Magellanic coastal population (pPA) displayed high morphological stability under salinity. The number and width of leaves were unaffected across treatments, and only a slight but significant reduction in leaf length was detected at 150 mM NaCl (14.31 ± 1.33 mm vs. 17.90 ± 0.93 mm at 0 mM; *p* < 0.05). Root length, biomass, and water content remained unchanged, indicating a high degree of morphological resilience.

The Andean inland population (pC) exhibited a distinct response. Leaf number increased significantly at 50 mM NaCl (20.27 ± 1.99) compared to the control (16.53 ± 0.97; *p* < 0.05), then declined at 150 mM (14.07 ± 0.66). Leaf dimensions and root length were not significantly affected. However, water content declined by 13% under severe salinity (from 82.40 ± 1.20 to 71.41 ± 2.65), suggesting reduced water retention capacity at high NaCl concentrations.

Associated with the morphological changes observed in all three populations, notable visual differences in plant habit were evident at 150 mM NaCl. In particular, the pA population exhibited leaf yellowing and loss of turgor, whereas pC plants maintained overall vigor (Supplementary Figure S1).

### 2.2. Physiological Responses to Salinity Stress

**Figure 1:**
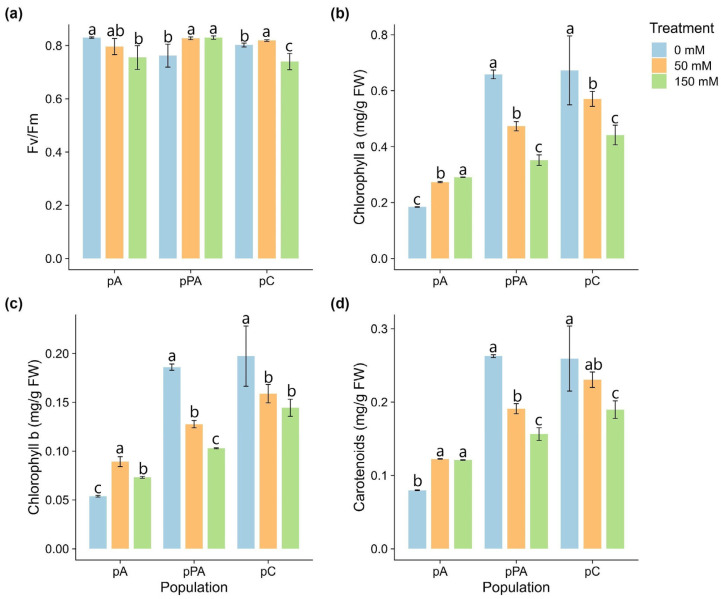
Photosynthetic parameters and pigment contents in *Colobanthus quitensis* plants from the Arctowski (pA), La Marisma (pPA), and Conguillío (pC) populations exposed to increasing NaCl concentrations. (**a**) Maximum quantum efficiency of PSII (Fv/Fm) (n = 5); (**b**) chlorophyll a; (**c**) chlorophyll b; and (**d**) carotenoids ((**b**–**d**), n = 3). Different letters indicate statistically significant differences between treatments within each population according to Tukey’s HSD post hoc test (*p* < 0.05).

Photosystem II efficiency (Fv/Fm) remained within optimal physiological values (>0.75) in all populations (Figure 1a). However, population-specific trends were evident. In pA, Fv/Fm significantly decreased at 150 mM NaCl. In pPA, it increased significantly from 50 mM onwards. In pC, Fv/Fm increased significantly at 50 mM compared to both control and 150 mM treatments, followed by a significant decline at 150 mM.

Chlorophyll a content varied across populations (Figure 1b). In pA, levels significantly increased with salinity, peaking at 150 mM. In pPA and pC, chlorophyll a significantly declined with increasing NaCl, with all comparisons being statistically different.

Chlorophyll b showed differential responses (Figure 1c). In pA, it increased under salinity, peaking at 50 mM. In pPA, a progressive and significant decrease occurred with increasing salinity. In pC, the decrease was significant only between control and salt-treated plants.

Carotenoids also responded differently (Figure 1d). In pA, levels increased significantly at both salinity levels. In pPA, carotenoids decreased progressively and significantly. In pC, values were stable from 0 to 50 mM but declined significantly at 150 mM.

### 2.3. Osmoprotective and Oxidative Stress Responses

**Figure 2:**
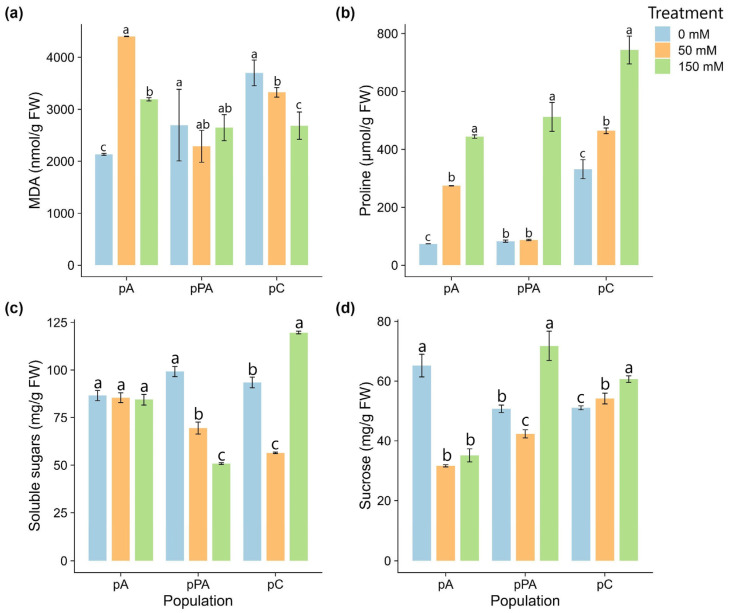
Biochemical parameters measured in leaves of *Colobanthus quitensis* plants from the Arctowski (pA), La Marisma (pPA), and Conguillío (pC) populations exposed to different NaCl concentrations. (**a**) Malondialdehyde (MDA) content; (**b**) Proline; (**c**) Total soluble sugars (TSS); (**d**) Sucrose content. Different letters indicate significant differences among treatments within each population according to Tukey’s HSD post hoc test (*p* < 0.05; n = 3).

Salinity induced population-specific oxidative and osmotic responses in *C. quitensis* (Figure 2). In pA, MDA levels doubled at 50 mM, then slightly decreased at 150 mM but remained above control. In pPA, MDA levels were stable across treatments. In pC, MDA significantly decreased at 150 mM, suggesting reduced oxidative damage under high salt.

Proline accumulation differed by population. In pA, it increased progressively, reaching 444.1 µmol g^−1^ FW at 150 mM. In pPA, a significant rise was observed only at 150 mM. In pC, proline accumulation was highest overall, peaking at 742.8 µmol g^−1^ FW at 150 mM, with significant increases at both salinity levels.

TSS levels remained stable in pA. In pPA, TSS declined significantly with increasing salinity. In pC, TSS declined at 50 mM but rose at 150 mM, exceeding control values.

Sucrose content decreased sharply in pA and pPA under salinity. In contrast, sucrose levels in pC remained statistically unchanged across treatments.

### 2.4. Antioxidant Enzyme Activity

**Figure 3:**
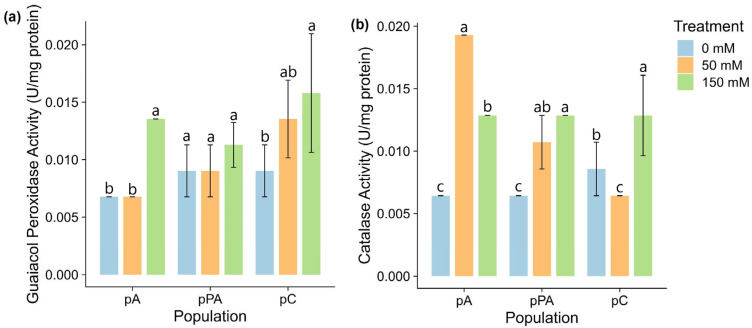
Enzymatic antioxidant activity in leaves of *Colobanthus quitensis* populations from Arctowski (pA), La Marisma (pPA), and Conguillío (pC) exposed to different NaCl concentrations. (**a**) Guaiacol peroxidase (GPx) activity. (**b**) Catalase (CAT) activity. Different letters indicate statistically significant differences between treatments within each population according to Tukey’s HSD post hoc test (*p* < 0.05; n = 3).

Antioxidant enzyme activity varied among populations under salinity (Figure 3). GPx activity remained unchanged in pA and pPA. In pC, it increased significantly at 150 mM, indicating inducible antioxidant activity under stress (Figure 3a).

CAT activity showed more pronounced variation (Figure 3b). In pA, it increased significantly at 50 mM and decreased slightly at 150 mM but remained above control. In pPA, CAT increased significantly only at 150 mM. In pC, CAT declined significantly at 50 mM and recovered at 150 mM to control levels.

### 2.5. Non-Enzymatic Antioxidant Responses

**Figure 4:**
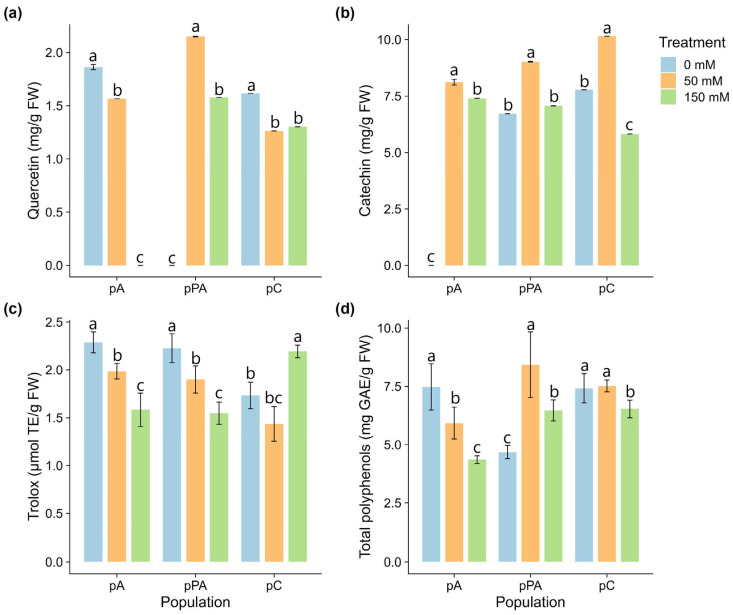
Non-enzymatic antioxidant responses in *Colobanthus quitensis* plants from the Arctowski (pA), La Marisma (pPA), and Conguillío (pC) populations subjected to different NaCl concentrations. (**a**) Quercetin content, (**b**) catechin content, (**c**) total antioxidant capacity (measured as Trolox equivalents), and (**d**) total polyphenol content. Bars represent mean values ± SE (n = 3). Different letters indicate statistically significant differences between treatments within each population, according to Tukey’s HSD post hoc test (*p* < 0.05).

Non-enzymatic antioxidant responses were population-specific (Figure 4). In pA, quercetin levels decreased significantly with salinity, becoming undetectable at 150 mM. In pPA, quercetin increased at 50 mM and declined at 150 mM but remained above control. In pC, a progressive decline was observed across treatments (Figure 4a). Catechin increased at 50 mM in all populations. At 150 mM, it decreased in pA and pC but remained unchanged in pPA (Figure 4b). Antioxidant capacity (Trolox equivalents) decreased significantly at 150 mM in pA and pPA. In pC, it declined at 50 mM but increased at 150 mM, surpassing control levels (Figure 4c). Total polyphenols remained stable in pA and pC. In pPA, they increased at 50 mM and slightly decreased at 150 mM (Figure 4d).

### 2.6. Integrative Multivariate Insights into Tolerance Mechanisms

**Figure 5:**
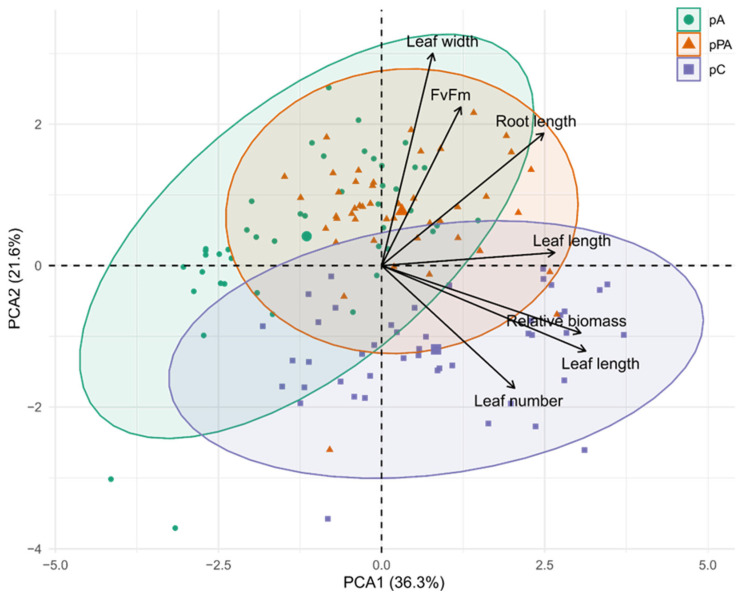
Principal component analysis (PCA) biplot based on morphological and physiological traits measured in *Colobanthus quitensis* plants exposed to different NaCl concentrations. Arrows represent the direction and magnitude of each variable’s contribution to the first two principal components (PC1 = 36.32%, PC2 = 21.62%), which together explain 57.94% of the total variance. Morphological traits included leaf length, leaf width, leaf number, root length, and relative biomass; physiological performance was represented by Fv/Fm. Colors indicate population of origin: green = Arctowski (pA), orange = La Marisma (pPA), and purple = Conguillío (pC). Ellipses represent 95% confidence intervals for each population.

PCA of morphological and physiological traits explained 57.94% of the total variance (PC1 = 36.32%, PC2 = 21.62%) (Figure 5). PC1 was associated with structural development, with major contributions from leaf length (25.36%), biomass (24.16%), and root length (18.25%). The pC population showed positive scores along PC1, suggesting superior growth maintenance under salinity. PC2 captured variation in water balance and photosynthetic efficiency, driven by leaf width (39.27%), Fv/Fm (21.93%), and water content (15.26%). The pA population clustered positively along PC2, indicating better photochemical and water status performance under stress.

**Figure 6:**
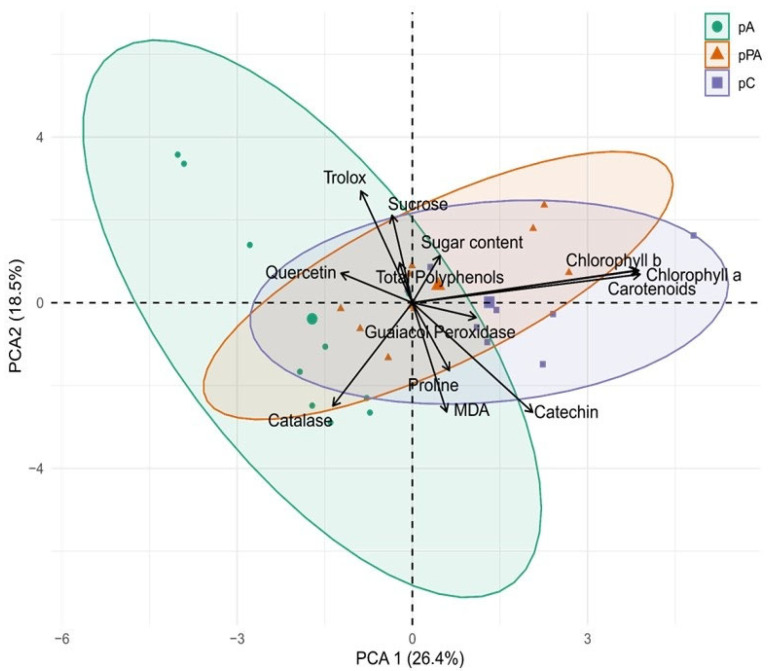
Principal component analysis (PCA) based on biochemical variables measured in *Colobanthus quitensis* plants from the Arctowski (pA), La Marisma (pPA), and Conguillío (pC) populations exposed to different NaCl concentrations. Arrows represent the direction and relative contribution of each biochemical variable to the first two principal components (PC1 = 26.36%, PC2 = 18.53%). Variables include chlorophyll a, chlorophyll b, carotenoids, malondialdehyde (MDA), catalase (CAT), guaiacol peroxidase (GPx), catechin, quercetin, total polyphenols, and antioxidant capacity (Trolox equivalents). Colors denote population origin: green = pA, orange = pPA, purple = pC. Ellipses indicate 95% confidence intervals for each population. All data were standardized prior to analysis. n = 3 per treatment.

Principal Component Analysis (PCA) based on biochemical variables revealed distinct clustering patterns among the three *C. quitensis* populations, reflecting their contrasting responses to salinity stress (Figure 6). Prior to analysis, data were standardized to ensure comparability across PCA of biochemical variables (Figure 6) explained 44.89% of total variance (PC1 = 26.36%, PC2 = 18.53%). PC1 was associated with photosynthetic pigments and antioxidant potential, with high contributions from carotenoids (27.3%), chlorophyll a (27.0%), and chlorophyll b (26.5%). Positive PC1 scores in pC suggested higher pigment accumulation and photoprotection.

PC2 was associated with antioxidant activity and oxidative stress markers, including Trolox (18.6%), catechin (17.9%), MDA (17.8%), and CAT (15.9%). pA showed positive association with PC2, indicating stronger non-enzymatic antioxidant activation. pPA clustered with enzymatic responses, such as CAT and GPx, suggesting distinct antioxidant strategies.

Together, these multivariate analyses provide strong evidence for population-specific salinity tolerance mechanisms in *C. quitensis*, emphasizing the value of integrating biochemical, physiological, and morphological traits. The robust and coordinated response of the Andean population (pC), despite the absence of marine influence, underscores the ecological and biotechnological relevance of non-coastal populations for resilience in extreme environments.

## 3. Discussion

Contrary to our initial hypothesis, predicting superior salinity tolerance in coastal populations, our findings revealed that the inland Andean population (pC) exhibits the most robust and integrated defense strategy. This unexpected result challenges the assumption that stress tolerance is solely driven by direct local selective pressures and instead suggests the involvement of more complex adaptive histories, possibly including exaptive or preadaptive mechanisms.

Understanding how plants respond to salinity, particularly through morphological development and antioxidant systems, is key to deciphering mechanisms of abiotic stress adaptation [19,20]. Such knowledge also supports the identification of genotypes with contrasting tolerance capacities, which are critical for ecological and biotechnological applications. In this context, our study provides novel evidence of population-specific salinity response strategies in *C. quitensis*, a vascular plant with a broad latitudinal and altitudinal distribution across South America and Antarctica. While the general resilience of this species to extreme environments has been previously acknowledged, this is the first integrated comparison of physiological, biochemical, and antioxidant responses across inland and coastal populations under standardized saline treatments. The observed variation aligns with the hypothesis that local environmental conditions have shaped distinct mechanisms of salt stress resilience [21], underscoring the potential of *C. quitensis* as a model system for studying adaptive stress responses in extremophile plants.

### 3.1. Morphological and Photosynthetic Adjustments

Salinity imposes multifaceted constraints on plant growth, making morphological traits critical indicators for assessing tolerance. Widely accepted selection criteria for salt-tolerant genotypes include shoot and root length, biomass accumulation, leaf production, and shoot area [22,23,24]. In our study, the Antarctic population (pA) displayed the most pronounced growth reductions under salinity, particularly in leaf number, root length, and total biomass. These impairments are consistent with the known effects of osmotic and oxidative stress on cellular expansion and metabolism [25].

In contrast, the Magellanic population (pPA) maintained stable growth even at 150 mM NaCl, suggesting morphological resilience potentially linked to its coastal habitat and historical exposure to periodic salt stress [15,17,18]. Notably, the inland Andean population (pC), originating from a high-altitude, non-saline ecosystem, exhibited only moderate inhibition of vegetative growth. This observation supports the possibility that adaptation to high-altitude abiotic stress confers a form of cross-tolerance to salinity [26]. However, this tolerance appears to be developmental stage-specific, as previous findings report a significant reduction in seed germination of pC under low salinity (50 mM NaCl) [18].

Photosynthesis is one of the primary processes affected by salt stress [27]. Although Fv/Fm is not a direct measure of carbon assimilation, it remains a widely accepted and non-destructive indicator of photoinhibition and the functionality of photosystem II. In *C. quitensis*, altered photosynthetic responses have been documented under diverse stressors, including cold, heat, and UV-B radiation [28,29,30], offering a reliable reference for evaluating photochemical efficiency under stress. However, future studies may incorporate complementary gas exchange or chlorophyll fluorescence parameters to further characterize the photosynthetic performance under salinity.

In our study, the decline in the Fv/Fm ratio observed in pA and pC at higher NaCl concentrations (Figure 1a) suggests early photoinhibition or partial damage to PSII photochemical efficiency, likely linked to oxidative stress [31]. Nevertheless, the general stability of Fv/Fm across all treatments indicates that PSII function was maintained, possibly through activation of photoprotective mechanisms such as carotenoid accumulation, which dissipates excess energy and protects photosystems from photodamage [32].

Ion imbalance is a common consequence of salt exposure, particularly due to Na^+^ accumulation and disruption of K^+^ homeostasis. This can lead to thylakoid membrane destabilization, pigment degradation, and reduced photosynthetic capacity [33]. These processes may explain the pigment declines observed in pC, and to a lesser extent in pPA. By contrast, pA displayed increased chlorophyll and carotenoid content under high salinity, an atypical but potentially adaptive response. This upregulation suggests an active photoprotective adjustment aimed at stabilizing PSII and preserving photosynthetic performance despite stress. Overall, the pigment dynamics and stable photochemical efficiency observed across populations indicate the deployment of distinct physiological strategies to maintain metabolic integrity under salt stress.

### 3.2. Osmotic Regulation and Membrane Protection

Malondialdehyde (MDA) is a widely recognized biomarker of lipid peroxidation, providing a reliable estimate of oxidative damage to cellular membranes under abiotic stress [34]. In our study, the Antarctic population (pA) was the only group to exhibit a significant increase in MDA levels under saline conditions, suggesting heightened susceptibility to oxidative stress and membrane destabilization (Figure 2a). In contrast, the Andean population (pC) displayed a marked decrease in MDA content at higher NaCl concentrations, indicating the activation of efficient protective mechanisms that mitigate lipid peroxidation. This contrasting pattern supports the hypothesis that plants adapted to high-altitude environments (such as pC) may possess inherently enhanced antioxidant and osmotic regulatory systems, enabling them to limit oxidative damage more effectively [35].

Proline is a key osmolyte and one of the most consistent biochemical markers of stress tolerance in plants. Its accumulation is commonly associated with the activation of multiple protective pathways that enhance survival under adverse conditions [36,37]. As a multifunctional molecule, proline contributes to protein and membrane stabilization, protects subcellular structures, and acts as a reactive oxygen species (ROS) scavenger under osmotic stress [38,39,40]. It also plays a protective role in maintaining photosynthetic function during salt exposure by limiting oxidative injury [41]. In our study, all three *C. quitensis* populations increased proline accumulation in response to salinity, reflecting a conserved osmotic adjustment mechanism. Notably, the pC population exhibited the highest proline levels at 150 mM NaCl (Figure 2b), suggesting a particularly robust response to osmotic stress. Due to its central role in stress resilience, proline metabolism has become a promising target in genetic improvement programs for salt-tolerant crops [42].

Alongside proline, variations in total soluble sugars (TSS) and sucrose content among populations provide additional insights into population-specific osmoprotective strategies shaped by native environmental pressures. The Magellanic coastal population (pPA) exhibited a consistent increase in sucrose levels across all salinity treatments (Figure 2d), suggesting a role for sucrose as a central osmotic regulator. Beyond serving as a carbon and energy reserve, sucrose contributes to osmotic balance and acts as an ROS scavenger, thus limiting oxidative damage under stress [43,44]. This sustained accumulation pattern may underlie the morphological stability of pPA under high salinity.

In contrast, the pC population showed a sharp increase in TSS at 150 mM NaCl (Figure 2c), in line with typical responses to osmotic stress. Similar sugar accumulation patterns have been previously reported in *C. quitensis* under copper toxicity [35] and cold stress [45]. Based on prior observations of sucrose predominance in *C. quitensis* under stress conditions, we evaluated sucrose separately to explore its specific contribution to osmotic adjustment and potential differential accumulation across populations. pC also displayed a significant increase in sucrose content under severe salinity (Figure 2d). The multifunctional nature of sucrose, via its metabolic, structural, and signaling functions, is crucial for maintaining osmotic homeostasis and reducing stress-induced damage, ultimately contributing to enhanced survival under extreme environmental conditions [46,47].

### 3.3. Antioxidant Defense: Enzymatic vs. Non-Enzymatic Strategies

Salinity stress, like other abiotic challenges, frequently triggers excessive production of reactive oxygen species (ROS), necessitating the activation of antioxidant defense systems to prevent oxidative damage. Among the enzymatic components, guaiacol peroxidase (GPx), a heme-containing enzyme, catalyzes the oxidation of aromatic substrates using hydrogen peroxide (H_2_O_2_), whereas catalase (CAT) decomposes H_2_O_2_ into water and oxygen [48]. These two enzymes were selected because of their central roles in H_2_O_2_ detoxification and their widespread use as markers of oxidative stress in plants [7,8]. As an extremophile, *C. quitensis* is hypothesized to rely on robust antioxidant mechanisms to withstand its native high-stress environments [49]. Previous research has documented variable enzymatic responses among *C. quitensis* ecotypes under different environmental stimuli, indicating high plasticity and acclimation capacity [13,50,51,52]. This includes coordinated GPx and CAT activation under warming conditions [53], reinforcing their central role in the oxidative stress response of this species. A potential limitation of our study is the use of fresh weight (FW) for the normalization of biochemical parameters. While this approach aligns with prior studies in *C. quitensis* [28,35] and avoids errors associated with variable tissue dehydration, it does not fully account for changes in dry matter content under stress. Future studies incorporating both FW- and DW-based normalization may provide more comprehensive insights, particularly when tissue availability permits.

In our study, salinity induced significant increases in both GPx and CAT activity across all populations, although the intensity, timing, and pattern of induction varied. GPx activity remained relatively stable overall, with a delayed yet marked increase in population pC at 150 mM NaCl, suggesting a role in long-term oxidative stress mitigation. In contrast, CAT exhibited more immediate and differentiated responses: population pA showed a strong early induction even at moderate salinity, whereas pPA and pC only activated CAT at higher salt concentrations. This pattern suggests that CAT may play a key role in the early detoxification of H_2_O_2_ in pA, while pC may prioritize delayed but coordinated antioxidant responses. Notably, the increase in GPx activity in pC coincided with elevated total antioxidant capacity at 150 mM NaCl (Figure 3), suggesting synergistic activation of enzymatic and non-enzymatic detoxification mechanisms [25]. These population-specific patterns align with previous reports on differential redox responses among *C. quitensis* ecotypes [54] and support the idea that distinct redox strategies have evolved in response to local environmental pressures [55].

Beyond enzymatic responses, non-enzymatic antioxidants, particularly flavonoids, play a fundamental role in protecting cellular structures under salinity stress. These compounds can directly scavenge ROS, inhibit pro-oxidant enzymes, and regulate stress signaling pathways [41]. In population pA, catechin levels increased significantly, while total polyphenol and quercetin contents declined, suggesting a shift in flavonoid composition under stress. The sharp decline in quercetin content under high salinity conditions suggests a limited capacity to sustain the pool of non-enzymatic antioxidants. Although pA plants activated enzymatic defenses early, particularly SOD and CAT, the concurrent decrease in quercetin may reflect an imbalance between ROS production and antioxidant replenishment. A plausible explanation is that quercetin was rapidly consumed in ROS detoxification and could not be regenerated efficiently, leading to oxidative damage and compromised growth. Alternatively, this decline may indicate a shift in metabolic priorities, where carbon allocation is diverted away from secondary metabolism (e.g., flavonoid biosynthesis) towards essential survival pathways under stress. These hypotheses warrant further investigation through the quantification of other phenolics and the expression analysis of key flavonoid biosynthetic genes. The observed pattern reinforces the idea that pA may rely predominantly on rapid enzymatic defenses rather than sustained non-enzymatic mechanisms to cope with episodic salinity events associated with marine spray. Catechin’s multifunctional antioxidant properties, including ROS scavenging and modulation of signaling cascades, are well documented [56]. In population pPA, both catechin and quercetin accumulated under stress, but without a corresponding increase in total antioxidant capacity. This decoupling between compound abundance and antioxidant performance has been reported previously in *C. quitensis* under UV-B and cold stress [57] and highlights the complexity of interpreting polyphenol profiles as direct indicators of physiological protection. The antioxidant potential of specific compounds depends not only on concentration but also on chemical structure, subcellular localization, and synergistic interactions [58]. Notably, quercetin is commonly synthesized under abiotic stress and displays strong ROS scavenging activity due to its hydroxyl-rich molecular structure [59].

Interestingly, in pC, both total polyphenol and flavonoid content decreased under high salinity, yet total antioxidant activity significantly increased at 150 mM NaCl. This suggests a more efficient use of available antioxidants or the contribution of alternative protective compounds. Among these, proline, abundant in pC under stress, has recognized metal-chelating and antioxidant properties [60,61]. In parallel, soluble sugars such as glucose and galactose can act as reducing agents contributing to antioxidant capacity (as measured by FRAP), as previously observed in *C. quitensis* under metal stress [35]. In pC, total antioxidant activity (FRAP) remained unchanged at moderate salinity but increased at 150 mM, diverging from the pattern seen in pA and pPA. This nonlinear behavior suggests that antioxidant mobilization in pC is triggered predominantly under severe stress. These results support the idea that pC may employ a highly regulated, threshold-based strategy to activate defense mechanisms only when necessary.

Collectively, these findings reveal that non-enzymatic antioxidant responses in *C. quitensis* are both compound- and population-specific. Population pA exhibited early signs of oxidative imbalance, with declining polyphenol and quercetin levels despite catechin accumulation, suggesting a limited capacity for sustained antioxidant protection. In contrast, pPA initiated a broader polyphenolic response, yet without proportional antioxidant reinforcement, indicating possible inefficiencies in redox control. The Andean population pC displayed a delayed but integrated strategy, characterized by increased total antioxidant capacity at the highest salinity level despite reduced flavonoid content, suggesting reliance on synergistic or alternative protective mechanisms.

While these results underscore the efficient antioxidant and osmotic regulation in pC, a critical knowledge gap remains regarding ion homeostasis. Future studies should incorporate ionomic profiling (e.g., Na^+^/K^+^ ratios, ion compartmentalization) to determine whether pC’s superior performance also involves enhanced Na^+^ exclusion or vacuolar sequestration, key traits for achieving full salinity tolerance [62]. Such insights would clarify whether pC relies solely on osmotic and antioxidant adjustments or has developed a multi-tiered defense strategy.

Taken together, these population-specific responses highlight the evolutionary divergence in stress adaptation mechanisms within *C. quitensis*, with pC emerging as a promising model for dissecting integrated tolerance strategies under extreme environments.

### 3.4. Ecological and Functional Implications

These findings of this study challenge the conventional assumption that coastal populations of *C. quitensis* inherently possess greater salinity tolerance. Remarkably, the pC population, despite its lack of marine influence, exhibited robust osmotic regulation and antioxidant defense mechanisms under salt stress. This observation supports the concept of exaptive tolerance, whereby traits originally evolved to withstand other abiotic stressors, such as cold temperatures, high UV radiation, and low atmospheric pressure at high altitudes, may confer cross-protection against salinity [13,18].

Conversely, the limited antioxidant efficiency observed in the pPA population, despite its accumulation of flavonoids and polyphenols, illustrates the complexity of using biochemical markers as direct proxies for stress tolerance. These discrepancies highlight the importance of evaluating functional outcomes, such as growth maintenance and integrated physiological responses, alongside molecular and biochemical indicators.

From a practical standpoint, the identification of genotypes such as pC, which maintain growth while coordinating osmotic and redox homeostasis under salinity stress, represents a promising opportunity for biotechnological applications and stress-resilient crop development. These results reinforce the value of *C. quitensis* as a model species for exploring adaptation strategies to multiple environmental constraints. To deepen our understanding of the underlying mechanisms, further studies incorporating transcriptomic, ionomic, and metabolic profiling are warranted.

Notably, population pC has consistently exhibited atypical physiological and phenological traits in previous common garden and germination studies [14,17,18], supporting the idea that it represents a highly specialized high-altitude ecotype. These distinctive features, combined with its integrated salinity response, reinforce its potential as a model system for studying cross-tolerance mechanisms in extremophile plants. While further studies are needed to evaluate whether other inland populations of *C. quitensis* share similar traits, the current findings underscore the functional relevance of pC for advancing our understanding of stress adaptation. Expanding these analyses to include transcriptomic, ionomic, and reproductive data could provide deeper insights into the evolutionary and ecological significance of this ecotype within the species’ broader distribution range.

### 3.5. Integrated Tolerance Profiles Among C. quitensis Populations

The integrated analysis of morpho-physiological, biochemical, and antioxidant responses to salt stress revealed three clearly differentiated tolerance profiles, shaped by native environments and adaptive potential. The Antarctic coastal population (pA) exhibited a stress-sensitive profile: despite increases in photosynthetic pigments and strong proline accumulation, salinity caused significant reductions in growth, water content, and sucrose levels, along with elevated MDA levels, particularly at moderate salinity. Although CAT activity increased at 50 mM NaCl, neither enzymatic nor non-enzymatic antioxidant defenses were sufficient to counteract oxidative damage under severe stress conditions.

In contrast, the Magellanic coastal population (pPA) maintained notable morphological stability under salt exposure, preserving leaf and root traits even at high salinity. However, this structural resilience was not fully supported by biochemical performance, as antioxidant capacity decreased and oxidative stress indicators suggested that redox balance might be compromised under prolonged exposure. This pattern may reflect a reliance on constitutive hydraulic or anatomical traits rather than strong inducible biochemical defenses.

The Andean inland population (pC) demonstrated an unexpectedly robust and coordinated response to salinity. This included enhanced proline and soluble sugar accumulation, limited growth inhibition, and delayed but significant activation of antioxidant mechanisms under high salinity. The maintenance of photosynthetic integrity, coupled with increased GPx activity and total antioxidant capacity at 150 mM NaCl, supports an efficient, possibly constitutive tolerance strategy, despite the absence of direct marine influence.

Together, these distinctive profiles underscore the functional diversity of *C. quitensis* and challenge the assumption that proximity to the marine environment necessarily confers higher salinity tolerance. The inland pC population exhibited a broader and more integrated stress response than its coastal counterparts, highlighting its ecological and biotechnological relevance as a model for salinity tolerance research.

Finally, the differential responses observed among *C. quitensis* populations reflect a high degree of local adaptation, with the Andean population (pC) displaying a particularly robust physiological and biochemical performance under high salinity, despite lacking marine influence in its native habitat. These results align with the concept of exaptive tolerance and support the notion that stress tolerance traits may arise in response to non-saline environmental pressures, such as high UV radiation, low temperatures, and seasonal drought. The distinct profile of pC, which has also exhibited atypical traits in previous studies [14,18,63,64], reinforces the importance of exploring intraspecific variation within *C. quitensis* across diverse habitats. Further studies in additional inland populations and under controlled conditions will be essential to validate these patterns and clarify the extent of functional diversity within the species.

**Figure 7:**
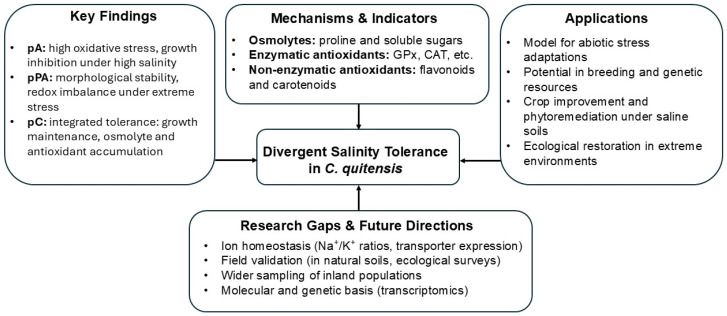
Conceptual framework of findings, future research directions, and applications of salinity tolerance in *Colobanthus quitensis*.

To frame the broader relevance of our findings, we present a conceptual diagram (Figure 7) that outlines the proposed future research directions. This includes molecular and physiological studies across additional *C. quitensis* populations, identification of candidate genes for salinity tolerance, and the exploration of biotechnological applications. This roadmap supports the positioning of *C. quitensis* as a valuable extremophile model for understanding stress adaptation mechanisms under climate change scenarios.

## 4. Materials and Methods

### 4.1. Experimental Design

#### 4.1.1. Plant Material and Growth Conditions

To analyze morphological, physiological, and biochemical responses, three populations of *C. quitensis* were selected from ecosystems spanning a latitudinal gradient. These included: an Antarctic population collected near the Polish Arctowski Station (62° S, hereafter pA), a population from a saltmarsh ecosystem in the city of Punta Arenas (53° S, hereafter pPA), and a population from Conguillío National Park (38° S, hereafter pC) (Table 2).

After field collection, individuals were transferred to growth chambers and vegetatively propagated with the support of the Colección Activa de Plantas Vasculares Antárticas (CAPVA) at the Laboratorio de Biotecnología y Estudios Ambientales (LABEA), Universidad de Concepción. Plants were cultivated under controlled conditions: temperature of 13 ± 2 °C, 16/8 h light/dark photoperiod, light intensity of 120 ± 20 µmol photons m^−2^ s^−1^, and 85–90% relative humidity. They were manually irrigated every two days and fertilized biweekly with Phostrogen (N.P.K. 13-10-27) at a concentration of 0.2 g·L^−1^. All populations were maintained under these conditions for six months and propagated vegetatively through three cycles prior to the onset of experimental treatments. During this period, populations were kept isolated to preserve genotype identity and avoid environmental preconditioning effects [14].

#### 4.1.2. Saline Treatments

For the salinity treatments, 190 individuals per population were randomly selected and acclimated for one month under the previously described growth conditions. After this acclimation period, individuals from each population were divided into three experimental groups, each subjected to a distinct salinity treatment: (1) control (0 mM NaCl), (2) low salinity (50 mM NaCl), and (3) high salinity (150 mM NaCl). The selected NaCl concentrations (50 and 150 mM) were chosen to represent a realistic range of salt stress intensities, informed by unpublished field data and previous studies on *C. quitensis* [17,18]. These concentrations reflect potential salinity conditions in Antarctic and Magellanic coastal soils influenced by marine spray and seasonal flooding. Although we lack soil salinity data for the Andean inland site (pC), its ecological contrast as a non-coastal population justifies its inclusion in the comparative analysis.

Saline solutions were applied by directly irrigating the substrate with the corresponding solution every two days, over a period of 21 days. Throughout the experiment, all growth conditions (i.e., temperature, photoperiod, light intensity, and relative humidity) were kept constant to ensure uniform stress application. At the end of the experiment, morphological, physiological, and biochemical variables were measured for each population under each treatment.

### 4.2. Morpho-Physiological Variables

To assess plant development under salinity treatment, morphological variables were recorded at the end of the experiment (day 21). For each population and treatment, ten individuals were randomly selected for measurement. The number of leaves per plant was recorded, and leaf dimensions (length and width, in mm) were measured manually using a digital caliper (Kamasa Tools, Enköping, Sweden; precision ±0.01 mm). Shoot length was determined by measuring the aerial tissue in its entirety, from the root–shoot junction (crown) to the tip of the longest leaf, reflecting the total shoot elongation. The primary root length (mm) was also measured separately for each plant.

Total biomass and water content were evaluated as additional morpho-physiological indicators. Ten complete aerial individuals per treatment were harvested and immediately weighed to obtain fresh weight (FW). Samples were then oven-dried at 60 °C for 72 h to constant weight and weighed again to obtain dry weight (DW). Total biomass was recorded as DW per plant (g), and water content (%) was calculated using the Formula (1):

Water content (%) = ((FW − DW)/FW) × 100(1)

All determinations were performed using ten biological replicates per treatment (n = 10).

### 4.3. Physiological Variables

The maximum photochemical efficiency of photosystem II (Fv/Fm) was measured at the end of the experiment (day 21) to evaluate the impact of salinity on the photosynthetic performance of *C. quitensis*. Measurements were conducted at the same time of day for all samples to ensure consistency. For each treatment and population, five individual plants were randomly selected, and three fully expanded, healthy leaves per plant were analyzed. Leaves were dark-adapted for 30 min prior to measurement using a Handy PEA fluorometer (Hansatech Instruments Ltd., King’s Lynn, UK). Fluorescence data were recorded under controlled growth chamber conditions. The Fv/Fm ratio was calculated, and the mean value per plant was used to determine treatment-level means for each population. Although the performance index (PI) was also measured, it showed no significant variation across treatments and was therefore excluded from further analysis.

At the end of the experiment (day 21), photosynthetic pigments were quantified as physiological indicators of photosynthetic function in response to salinity. For each treatment and population, three biological replicates were prepared by randomly sampling 200 mg of fresh leaf tissue per replicate. Samples were immediately macerated in liquid nitrogen and extracted with 15 mL of 80% (*v*/*v*) acetone, following the protocols of [65]. The extract was filtered through qualitative filter paper and adjusted to a final volume of 20 mL with 80% acetone. Absorbance was measured at 470, 646, and 663 nm using a GENESYS 10s UV-VIS spectrophotometer (Thermo Scientific, Waltham, MA, USA), with 80% acetone as blank. Chlorophyll a (Chl a) concentration was calculated using the following equation: (12.21 × Abs663 − 2.81 × Abs646); chlorophyll b (Chl b) using the following equation: (20.13 × Abs646 − 5.03 × Abs663); and carotenoid content using the formula: (1000 × Abs470 − 3.27 × Chl a − 104 × Chl b)/229). Results were expressed as milligrams of pigment per gram of fresh weight tissue (mg g^−1^ FW).

### 4.4. Biochemical Variables

At the end of the experiment (day 21), aerial fresh plant tissue samples from each population and treatment were harvested for biochemical analysis. Plants were gently washed with sterile distilled water to remove substrate residues, blotted dry with sterile absorbent paper, and stored at −80 °C until analyses were made. Due to the small size of *C. quitensis* individuals, biochemical analyses were restricted to aerial tissues. Root biomass was insufficient for replicate sampling without compromising plant viability. Also, biochemical parameters were normalized to fresh weight (FW), in accordance with established protocols for *C. quitensis* [28,35].

#### 4.4.1. Lipid Peroxidation

Lipid peroxidation was quantified as malondialdehyde (MDA) content following the method in [66]. For each treatment and population, three biological replicates were prepared by grinding 200 mg of aerial tissue in liquid nitrogen and homogenizing the sample in 1 mL of 0.5% (*v*/*v*) trichloroacetic acid (TCA). After centrifugation at 5300 rpm for 20 min, 0.5 mL of the supernatant was mixed with 2 mL of 0.5% (*w*/*v*) thiobarbituric acid (TBA) in 20% TCA. The mixture was incubated at 96 °C for 30 min, then cooled on ice to stop the reaction. Absorbance was measured at 532 and 600 nm using a GENESYS 10s UV-VIS spectrophotometer, and MDA content was calculated using the extinction coefficient (155 mM^−1^ cm^−1^) and expressed as nmol MDA per gram of fresh weight.

#### 4.4.2. Proline Content

Proline was quantified according to [67]. For each treatment and population, three biological replicates were prepared by grinding 250 mg of aerial tissue in liquid nitrogen and homogenizing the sample in 1.25 mL of 3% sulfosalicylic acid. After filtration, 0.5 mL of the extract was mixed with 0.5 mL of glacial acetic acid and 0.5 mL of ninhydrin. The mixture was incubated at 100 °C for 1 h, then cooled on ice. One milliliter of toluene was added, and the chromophore-containing upper phase was measured at 520 nm. Proline concentration was determined using a standard calibration curve (0–9 µg mL^−1^) and expressed as µg per gram of fresh weight.

#### 4.4.3. Total Soluble Sugar Content

Total soluble sugars were determined using a modified resorcinol method [68]. For each treatment and population, three biological replicates were prepared by extracting 200 mg of aerial tissue with 10 mL of 86% (*v*/*v*) ethanol at 60 °C for 30 min. After centrifugation (12,000 rpm, 10 min), 100 μL of the supernatant was mixed with 1.75 mL of 37% HCl, 250 μL of 1% (*w*/*v*) resorcinol, and 500 μL of distilled water. The mixture was incubated at 80 °C for 8 min, and absorbance was measured at 520 nm using pure sucrose as a standard.

#### 4.4.4. Sucrose Content

Sucrose concentration was measured using a Shimadzu Prominence high-performance liquid chromatography (HPLC) system coupled to an Applied Biosystems/MDS Sciex 3200 Qtrap Mass Spectrometer (Shimadzu Corporation, Tokyo, Japan), based on [69]. For each treatment and population, three biological replicates of 100 mg of aerial tissue were extracted in aqueous ethanol 80% (*v*/*v*) and stirred for 24 h. Samples were separated using an Agilent 1200 HPLC system equipped with a Zorbax carbohydrate column (4.6 × 150 mm, 5 μm) and a refractive index detector. The mobile phase consisted of acetonitrile: water (75:25 *v*/*v*) at a flow rate of 1 mL min^−1^. Quantification was performed using pure sucrose (Merck KGaA, Darmstadt, Germany) as a standard.

### 4.5. Enzyme Activity Analysis

Enzymatic antioxidant activities were determined from aerial tissue of plants collected at the end of the experiment (day 21). For each treatment and population, three biological replicates of 100 mg of aerial tissue were ground in liquid nitrogen and homogenized in 1.5 mL of extraction buffer containing 100 mM potassium phosphate (pH 7.4), 0.1% (*w*/*v*) EDTA, and 1% (*w*/*v*) polyvinylpyrrolidone (PVP). The homogenate was centrifuged at 10,000× *g* for 20 min at 4 °C, and the resulting supernatant was used for both protein quantification and subsequent enzymatic assays. All determinations were performed immediately after extraction to preserve enzyme activity.

#### 4.5.1. Protein Quantification

Total soluble protein content in the enzymatic extracts was determined using the Bradford assay [70]. An aliquot of 50 µL of the enzyme extract was mixed with 1.5 mL of Bradford reagent (Bio-Rad, Hercules, CA, USA), and absorbance was measured at 595 nm after 10 min of incubation at room temperature. Bovine serum albumin (BSA; Sigma-Aldrich, St. Louis, MO, USA) was used as the standard for calibration. Protein concentration was expressed as mg of BSA equivalents per mL of extract, and all enzyme activities were normalized accordingly.

#### 4.5.2. Guaiacol Peroxidase (GPx) Activity

GPx activity was measured according to [71]. The reaction mixture (3 mL) contained 2.8 mL of 100 mM potassium phosphate buffer (pH 7.0), 50 μL of 12 mM guaiacol, 50 μL of 100 mM H_2_O_2_, and 100 μL of enzymatic extract. The increase in absorbance at 470 nm was recorded at 10 s intervals for 10 min using a spectrophotometer (GENESYS 10s UV-VIS, Thermo Scientific, Waltham, MA, USA). Enzyme activity was expressed as units per minute (U min^−1^), where one unit corresponds to the formation of 1 μmol of tetraguaiacol per minute.

#### 4.5.3. Catalase (CAT) Activity

CAT activity was determined following the method of [72]. The reaction mixture consisted of 1.78 mL of 100 mM potassium phosphate buffer (pH 7.5), 100 μL of 100 mM H_2_O_2_, and 120 μL of enzymatic extract. The decomposition of H_2_O_2_ was monitored by the decrease in absorbance at 240 nm, measured every 10 s for 30 min in a quartz cuvette. Enzyme activity was expressed as units per minute (U min^−1^), where one unit corresponds to the decomposition of 1 μmol of H_2_O_2_ per minute.

### 4.6. Antioxidant Activity and Phenolic Compounds

To evaluate antioxidant capacity and phenolic compound profiles, aerial tissues were collected at the end of the experiment (day 21), dried, and processed for ethanolic extraction. For each treatment and population, three biological replicates of 100 mg of dry aerial tissue were extracted with 3 mL of 70% (*v*/*v*) ethanol. Samples were incubated for 12 h at room temperature in an orbital shaker (LOS F-17, Labtron Equipment Ltd., Sheffield, UK), then filtered through 0.45 µm nylon syringe filters (Corning, Corning, NY, USA). Extracts were stored at −18 °C until further analysis. It is important to note that total polyphenol content (Section 4.6.1) and FRAP antioxidant capacity (Section 4.6.3) were determined from this ethanolic extract, while phenolic compound profiling (Section 4.6.2) was conducted independently using methanolic extracts prepared from separate biological replicates, due to methodological and instrumental requirements of the collaborating laboratory.

#### 4.6.1. Total Polyphenols Content

Total polyphenol content was determined using the Folin–Ciocalteu method. In a 96-well plate, 100 µL of extract (diluted 1:10, *w*/*v* in 85% ethanol) was mixed with 50 μL of Folin–Ciocalteu reagent. After 5 min of incubation at room temperature, 150 μL of 20% (*w*/*v*) sodium carbonate and 700 μL of ultrapure water were added. The mixture was incubated for 2 h at room temperature in dark. Absorbance was measured at 760 nm using a Multiskan FC plate reader (Thermo Fisher Scientific Inc., Waltham, MA, USA). Gallic acid was used as the standard, and results were expressed as mg of gallic acid equivalents per g of dry weight (mg GAE g^−1^ DW).

#### 4.6.2. Phenolic Compound Profile

Individual phenolic compounds were identified and quantified using a targeted analysis approach based on the retention times and spectral characteristics of commercial standards. For each treatment and population, three biological replicates of 100 mg of dry aerial tissue were extracted with 90% (*v*/*v*) methanol and agitated overnight at room temperature. The analysis was conducted on a Shimadzu Prominence HPLC system coupled to an Applied Biosystems/MDS Sciex 3200 Qtrap Mass Spectrometer (Shimadzu Corporation, Tokyo, Japan), following [73] with modifications. Extracts were analyzed using a Nexera Lite HPLC system (Shimadzu Corporation, Kyoto, Japan) equipped with a ZORBAX Eclipse XDB-C18 column (4.6 × 150 mm, 5 μm; Agilent Technologies, Santa Clara, CA, USA) maintained at 37 °C. The mobile phase consisted of acidified acetonitrile and acidified distilled water, with a flow rate of 0.5 mL min^−1^.

The identification and quantification focused on key phenolic compounds, specifically quercetin and catechin, using their respective commercial standards for calibration and confirmation. Although apigenin was also included in the initial screening, the data obtained were inconsistent and therefore excluded from further analysis. Only compounds with consistent and reproducible detection across all replicates were reported in the results.

#### 4.6.3. Antioxidant Capacity Determination (FRAP Assay)

The total antioxidant capacity was determined using the Ferric Reducing Antioxidant Power (FRAP) assay, following the method of [74] with minor modifications. In a 96-well microplate, 10 μL of each ethanolic extract were mixed with 290 μL of freshly prepared FRAP reagent (300 mM acetate buffer pH 3.6, 10 mM TPTZ in 40 mM HCl, and 20 mM FeCl_3_·6H_2_O in a 10:1:1 ratio). After 30 min of incubation at 37 °C, absorbance was measured at 593 nm using a Multiskan FC microplate reader (Thermo Fisher Scientific Inc., Waltham, MA, USA). Antioxidant capacity was expressed as mg of Trolox equivalents per g of dry weight (mg TE g^−1^ DW), based on a standard curve.

### 4.7. Statistical Analysis

To evaluate treatment effects and identify response patterns associated with salinity treatments in *C. quitensis*, both univariate and multivariate statistical approaches were employed. All analyses were conducted using R software (v.4.3.0) [75]. To evaluate the effects of salinity treatment (NaCl) on the morphological, physiological and biochemical responses of plants, one-way analysis of variance (ANOVA) models were independently applied to each response variable for each population (pA, pPA, and pC) using the aov() function in R. Model assumptions of normality and homogeneity of variance were assessed prior to analysis. Normality of residuals was visually inspected using Q-Q plots, while homogeneity of variance was evaluated using residual vs. fitted value plots and formally tested with Levene’s test, as implemented in the car package in R [76]. When significant treatment effects were detected (*p* < 0.05), pairwise differences among treatments were determined using Tukey’s Honest Significant Difference (HSD) post hoc test. Data visualization, including bar plots with standard errors, treatment comparison, and multivariate outputs, was performing using ggplot2 package [77], with figure layout managed by cowplot [78].

To explore co-variation among morphological, physiological and biochemical variables, Principal Component Analysis (PCA) was conducted on standardized variables (mean = 0, standard deviation = 1). The analysis was performed using the PCA() function from the FactoMineR package [79], and visualization of biplots, variable contributions, and clustering were generated using the factoextra package [80]. Ninety-five % confidence ellipses were added to highlight potential groupings by treatment and population.

## 5. Conclusions

This study demonstrates that *C. quitensis* exhibits population-specific responses to salinity stress, shaped by the environmental conditions of their native habitats. The Antarctic population (pA) showed early activation of antioxidant defenses but experienced significant growth inhibition and oxidative damage. The Magellanic coastal population (pPA) maintained morphological integrity yet lacked sustained redox regulation under high salinity. In contrast, the Andean inland population (pC) displayed a robust and coordinated tolerance strategy, integrating growth maintenance, osmolyte accumulation, and activation of both enzymatic and non-enzymatic antioxidant mechanisms.

These findings underscore the underestimated resilience of non-coastal *C. quitensis* populations and highlight the importance of local adaptation in shaping plant responses to abiotic stress. The identification of divergent tolerance strategies among geographically distinct populations contributes to a deeper understanding of the species’ ecological plasticity and supports its use as a model for investigating stress adaptation in extremophile plants.

Further research should aim to elucidate the molecular and genetic foundations of these differential responses and assess whether similar patterns occur across other inland populations. The observed resilience of the pC population may hold biotechnological potential for crop improvement or phytoremediation strategies in saline-affected environments, especially under climate change scenarios. This resilience likely arises from the coordinated activation of osmoprotective and antioxidant mechanisms, traits that could be harnessed through breeding, genetic engineering, or physiological priming to develop crops with enhanced tolerance to salinity. Such traits may also inform the selection of species for phytoremediation programs in salt-affected soils under future climate change scenarios.

## Figures and Tables

**Table 1:** Morphological and whole-plant performance measurements of *Colobanthus quitensis* populations from Arctowski (pA), La Marisma (pPA), and Conguillío (pC) exposed to three salt concentrations. Different letters indicate significant differences between means according to Tukey’s HSD post hoc test (*p* < 0.05; n = 10).

Variable	Treatment	pA	pPA	pC
Number of leaves	0 mM	18.67 ± 0.93 ^a^	12.67 ± 1.16 ^a^	16.53 ± 0.97 ^ab^
	50 mM	11.47 ± 1.02 ^b^	10.4 ± 0.89 ^a^	20.27 ± 1.99 ^a^
	150 mM	8.87 ± 0.50 ^b^	9.67 ± 0.57 ^a^	14.07 ± 0.66 ^b^
Leaf width (mm)	0 mM	1.42 ± 0.52 ^a^	1.19 ± 0.04 ^a^	0.76 ± 0.03 ^a^
	50 mM	1.14 ± 0.06 ^b^	1.32 ± 0.05 ^a^	0.86 ± 0.03 ^a^
	150 mM	1.01 ± 0.06 ^b^	1.28 ± 0.04 ^a^	0.81 ± 0.04 ^a^
Leaf length (mm)	0 mM	13.02 ± 0.63 ^a^	17.9 ± 0.93 ^ab^	23.87 ± 1.32 ^a^
	50 mM	10.36 ± 0.75 ^b^	19.9 ± 1.79 ^a^	25.11 ± 1.58 ^a^
	150 mM	8.86 ± 0.38 ^b^	14.31 ± 1.33 ^b^	24.10 ± 1.36 ^a^
Root length (mm)	0 mM	45.90 ± 2.35 ^a^	60.89 ± 3.86 ^a^	57.88 ± 3.68 ^a^
	50 mM	40.39 ± 2.93 ^a^	61.04 ± 2.93 ^a^	62.81 ± 7.70 ^a^
	150 mM	31.15 ± 1.49 ^b^	62.24 ± 3.82 ^a^	49.96 ± 4.67 ^a^
Biomass (g)	0 mM	1.02 ± 0.10 ^a^	1 ± 0.15 ^a^	0.98 ± 0.18 ^a^
	50 mM	0.62 ± 0.09 ^b^	1.05 ± 0.08 ^a^	1.24 ± 0.13 ^a^
	150 mM	0.43 ± 0.07 ^b^	0.87 ± 0.09 ^a^	1.28 ± 0.14 ^a^
Water content (%)	0 mM	81.16 ± 1.67 ^a^	81.94 ± 1.15 ^a^	82.40 ± 1.20 ^a^
	50 mM	70.65 ± 3.63 ^b^	81.93 ± 1.83 ^a^	80.38 ± 1.21 ^a^
	150 mM	73.26 ± 2.81 ^ab^	81.77 ± 1.31 ^a^	71.41 ± 2.65 ^b^

**Table 2:** Characteristics of the collection sites of the *Colobanthus quitensis* populations.

Population	Origin	Geographical Location	Altitude (m.a.s.l.)	Marine Influence
pA	Polish Antarctic Station H. Arctowski, Admiralty Bay, King George Island	62°09′ S; 58°28′ W	3–23	Regularly exposed to sea spray due to strong coastal winds
pPA	Punta Santa María Sector, South of Punta Arenas, Chile	53°22′ S; 70°58′ W	1–3	Subject to seawater flooding during high tides
pC	Conguillío National Park, La Araucanía, Chile	38°36′ S; 71°36 W	2575	No marine influence

## Data Availability

The data will be available upon request from the corresponding author.

## Supplementary Materials

The following supporting information can be downloaded at: https://www.mdpi.com/article/10.3390/plants14203116/s1, Figure S1: Growth of *Colobanthus quitensis* plants from the Arctowski (pA), La Marisma (pPA), and Conguillío (pC) populations after exposure to 0 (Control), 50, and 150 mM NaCl for 21 days.
