# Supplementary figures and images for: Population-Specific Salinity Tolerance in the Extremophile Colobanthus quitensis: Evidence of Adaptive Plasticity
Source: Plants (Basel). 2025 Oct 10;14(20):3116. doi: 10.3390/plants14203116 (PMC12566950; doi:10.3390/plants14203116)

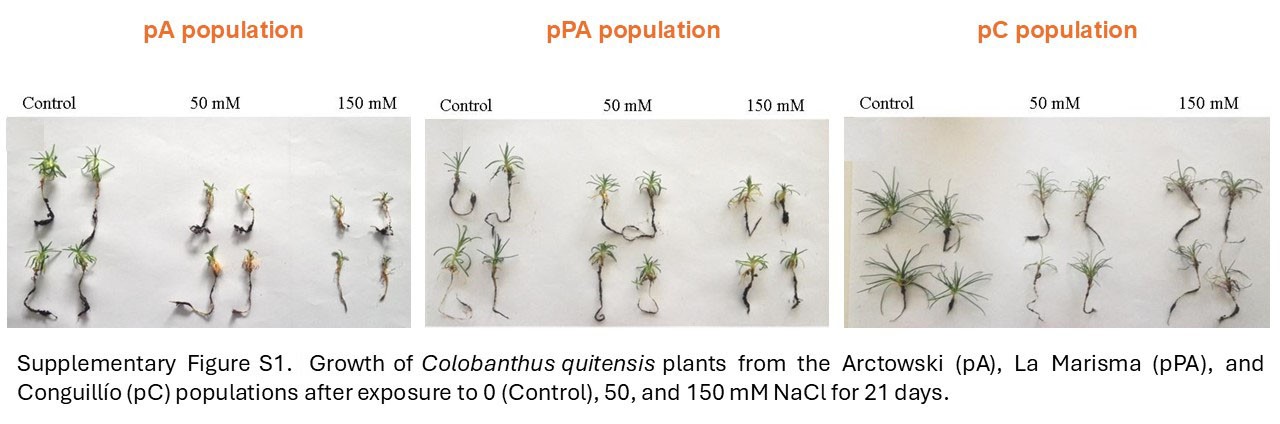

Supplement: Supplementary file 1 [file plants-14-03116-s001.zip › plants-3875899-supplementary.jpg]
